# Periodontal Diseases and Pregnancy: Knowledge and Clinical Practice Habits of French Midwives

**DOI:** 10.3290/j.ohpd.b3680323

**Published:** 2022-12-13

**Authors:** Solen Novello, Marion Pailleau, Pierre Le Dévéhat, Sylvie Jeanne

**Affiliations:** a Associate Professor, Faculty of Dentistry, University of Rennes 1, and Department of Periodontology, University Hospital of Rennes, France. Conceptualisation, formal analysis, methodology, supervision, wrote original draft, reviewed and edited the manuscript.; b Doctor of Dental Surgery, Faculty of Dentistry, University of Rennes 1, Rennes, France. Conceptualisation, data curation, formal analysis, methodology, wrote original draft.; c Doctor of Dental Surgery, Faculty of Dentistry, University of Rennes 1, Rennes, France. Conceptualisation, data curation, formal analysis, methodology, wrote original draft.; d Professor, Faculty of Dentistry, University of Rennes 1, and Department of Periodontology, University Hospital of Rennes, France. Conceptualisation, supervision, reviewed and edited the manuscript.

**Keywords:** adverse pregnancy, midwifery education, oral health, periodontal disease, pregnancy, prevention, outcomes

## Abstract

**Purpose:**

Several studies have demonstrated the role of periodontal disease as risk factor of adverse pregnancy outcomes, including preterm birth, low birthweight and pre-eclampsia. As such, midwives can play an essential role in prevention and early screening as the preferred intermediary of pregnant women. The purpose of this study was to assess the knowledge, training and daily practice habits of midwives to determine if they fulfill their role in oral health prevention.

**Materials and Methods:**

A questionnaire was sent by e-mail to practicing midwives and fifth-year midwifery students in the Brittany region of France. Data were collected online and descriptive data analyses were conducted.

**Results:**

A total of 192 practicing midwives and 13 students participated in the survey. The results showed that the majority of midwives were not familiar with the correlation between periodontal disease and adverse pregnancy outcomes and did not implement screening and prevention to a sufficient extent.

**Conclusion:**

The explanation for this lack of knowledge seems to come from the initial training, since the topic of oral health is almost never discussed during midwives’ studies. Most agreed they needed more training on periodontal disease and adverse pregnancy outcomes. Improving and integrating oral health education into the midwife academic curriculum can enhance midwives’ engagement in oral health.

Periodontal diseases are multifactorial inflammatory diseases characterised by the progressive and irreversible destruction of the tooth-supporting tissues.^36^ It is a dysbiotic disease, associated with an alteration in the abundance or influence of individual species within the polymicrobial community. Periodontal dysbiosis is associated with disruption of tissue homeostasis.^8,15^ The microbial communities interact with the immune and inflammatory response of the host, fundamentally governed by environmental and acquired risk factors of the individual, as well as host genetics. In some individuals, this leads to a deregulated response characterised by exacerbated inflammation, resulting in periodontal supportive-tissue destruction.^18,19^ In its most advanced forms, periodontitis manifests itself as tooth mobility that can go as far as spontaneous exfoliation of the teeth. Beyond their oral consequences, periodontal diseases have a proven impact on general health.^28^ This is largely related to the translocation of bacteria from the oral cavity to peripheral organs, and to increased levels of systemic inflammation.^21^ It is known that chronic inflammation originating from the oral cavity influences the pathogenesis of diseases at the systemic level.^14^ Conversely, systemic diseases can promote susceptibility to periodontitis by increasing the inflammatory burden of the periodontium or by modulating the periodontal microbiome.^41^

During pregnancy, there are many risk factors for complications, and periodontal disease is one of them. Several studies have highlighted the bidirectional link between periodontitis and adverse pregnancy outcomes, such as preterm birth, pre-eclampsia or low birth weight.^3,7,12,33,39^ The main hypothesis linking these two conditions are the hematogenous dissemination of periodontopathogenic bacteria and their components from the oral cavity to the feto-placental unit, and the continuous release of inflammatory mediators.^12,20^ The influence of the oral microbiota is not confined to this location, and bacteria associated with the oral cavity have been detected in many distant organ sites.^39^ Up to 75% of women develop gingivitis during pregnancy,^31^ probably due to a shift in the oral microbiome, with higher amounts of *Porphyromonas gingivalis* and *Aggregatibacter actinomycetemcomitans*.^5^ Elevated concentrations of estrogen and progesterone also play a role in inducing vasodilation, exacerbating the inflammatory response and altering the immune response, making the pregnant woman more likely to develop or worsen pre-existing periodontitis.^23,34^

Periodontitis is a direct consequence of untreated gingivitis. It can be treated and prevented through early intervention, oral health education and antenatal screening.^31^ International guidelines in the US, the UK and Europe encourage these approaches early in pregnancy.^22,30,38^ To this end, monitoring and maintaining periodontal health in pregnant patients represent an opportunity for interprofessional collaboration in health care to improve patient outcomes.

More and more women are turning to midwives for their pregnancy follow-up. As a preferred intermediary, they play an important role in the prevention and detection of risk factors of adverse pregnancy outcomes. They can inform patients accordingly, provide orientation, and reinforce the follow-up when periodontal disease markers are present from the beginning of the pregnancy. In France, 47% to 56% of women do not consult a dentist during their pregnancy.^35,43^ When they have, only 6% of them were referred by a health professional involved in monitoring their pregnancy.^24^

The low rate of dental visits and women referred by their midwife during pregnancy leads us to question their knowledge and training in the oral field, as well as the possible consequences on pregnancy. The aim of this study was to evaluate the knowledge of midwives from a Breton population about periodontal diseases and their implication in adverse pregnancy outcomes.

## Materials and Methods

### Study Design and Protocol

This study was conducted using an online questionnaire in French, edited through Google Forms. It was distributed from April 2021 to June 2021 to 595 midwives and 55 fifth-year midwifery students. The questionnaire was divided into two parts. The first part collected personal information in order to establish a profile of the respondent, and was designed to maintain strict anonymity. The second part was divided into three sections, asking respondents about their practice habits, knowledge and training. Prior to its distribution, a protocol for this study was written, along with an information note for the participants. This protocol was approved by the Ethics Committee of the University Hospital of Rennes (ref: 21.188).

### Population

The studied population consisted of practicing midwives, whatever their mode of practice was, and fifth-year midwifery students, in the Brittany region. Concerning hospital practice, the level of the maternity ward was specified (1 for risk-free pregnancy, 2 for moderate-risk pregnancy and 3 for high-risk pregnancy). Retired midwives were excluded from the survey.

### Data Analysis

Data were analysed using PC-based software. Analyses were performed using R (version 4.1.0)^37^ and Epi Info (version 7.2.4.0).^11^ The qualitative variables were compared using the Χ^2^ test via Epi Info if the conditions were met; otherwise the Fisher’s exact test was used via R. A p-value < 0.05 was considered statistically significant.

## Results

### Demographic Data

Demographic data are presented in Table 1. A total of 192 out of 595 practicing midwives (32%) and 13 out of 55 students (24%) answered the questionnaire. The population was between 22 and 62 years old. 95% of the participants were women. Of these women, 68.7% had children and 20.9% had oral problems during pregnancy. Most participants (71.3%) practiced in a hospital setting, especially in a level-2 maternity ward.

**Table 1:** Demographic data

Demographic data	Population n (%)
Gender (N = 205)	
Male Female	10 (4.9) 195 (95.1)
Study location (N = 205)	
Rennes Brest Nantes Abroad Other	92 (45.1) 35 (17.2) 12 (5.9) 14 (6.9) 52 (24.9)
Employment status (N = 205)	
Practicing midwife Midwifery student (fifth year)	192 (93.7) 13 (6.3)
Years of experience as a midwife (N = 192)	
< 5 5–10 11–15 16–20 ˃ 20	41 (21.4) 37 (19.3) 32 (16.7) 26 (13.5) 56 (29.2)
Mode of practice (N = 192)	
Hospital Self-employed Territorial communities Other	137 (71.3) 45 (23.4) 6 (3.1) 4 (2.1)
Level of the maternity ward (N = 137)	
1 2 3	28 (20.4) 74 (54.0) 35 (25.5)
Do you have children? (N = 195)	
Yes No	134 (68.7) 61 (31.3)
If yes, did you have any oral problems during your pregnancy? (N = 134)	
Yes No	28 (20.9) 106 (79.1)

Practicing midwives were divided into several groups according to their professional experience. Each group comprised between 13.5% and 21.4% of the sample, with the exception of midwives with more than 20 years of practice, who represented a larger proportion (29.2%).

The majority of midwives and students in the sample had studied in Rennes (45.1%) and Brest (17.2%).

### Practice Habits

40% of the participants never provided oral-health-related information during a consultation, and 45% did so sometimes. Of the 13 students, nine never did, four sometimes. There was a statistically significant difference between hospital and self-employed midwives (p = 0.00003), with those working in private practice raising the subject more often during their consultations. No statistically significant difference was found when comparing the different levels of maternity wards (p = 0.07) (Table 2).

**Table 2:** Oral health concerns during consultations, according to midwives’ mode of practice (mode “other”excluded)

		n	Never	Sometimes	Often	Always	p-value
Self-employed practice n (%)		45	7 (15.6)	21 (46.7)	10 (22.2)	7 (15.5)	0.00003*
Hospital practice n (%)		137	64 (46.7)	65 (47.5)	7 (5.1)	1 (0.7)	
	Level 1	28	8	18	2	0	0.07
	Level 2	74	41	31	2	0	
	Level 3	35	15	16	3	1	
Territorial communities n (%)		6	0	3 (50.0)	3 (50.0)	0	
Total n (%)	188		71 (37.8)	89 (47.3)	20 (10.6)	8 (4.3)	

*p < 0.05. n = number of respondents related to demographic characteristics.

Midwives who had children were more likely to discuss oral health in their consultations (p = 0.0025). However, there was no statistically significant difference between those who had experienced oral health problems during pregnancy and those who had not (p = 0.32). There was also no difference regardless of where the midwives were trained (p = 0.23).

83.9% of midwives were aware that health insurance covers 100% of the cost of a preventive oral exam for pregnant women. However, 23.9% never advised their patients to make a dental appointment; 46.3% sometimes did.

Only 19 of the 205 midwives performed an oral health assessment to identify the risk of infection in patients hospitalised with high-risk pregnancy. Of these, four were self-employed, 14 were hospital-based and one was at another facility.

71.7% of had encountered a dental problem or issue during their practice. This percentage naturally increases with practice time, with 89% of midwives practicing for more than 20 years responding positively to the question. Of those who answered “yes”, half (49.7%) felt they were able to respond and/or identify the problem. The vast majority (86.4%) of participants answered “mostly yes” (48.8%) or “mostly no” (37.6%) to the question of whether they felt comfortable giving oral hygiene advice to their patients.

These data are presented in Table 3.

**Table 3:** Practice habits

Practice habits	Population n (%)
During a consultation, do you provide oral-health-related information to your pregnant patients? (N = 205)	
Never Sometimes Often Always	82 (40) 93 (45.4) 21 (10.2) 9 (4.4)
Do you refer your patients to their dentist for a check-up during their pregnancy? (N = 205)	
Never Sometimes Often Always	49 (23.9) 95 (46.3) 36 (17.6) 25 (12.2)
Of your patients hospitalised for a high-risk	
pregnancy, do you perform an oral health assessment to identify the risk of infection? (N = 205) Never Sometimes Often Always	186 (90.7) 18 (8.8) 1 (0.5) 0 (0)
Do you feel comfortable giving oral hygiene advice to your patients (duration, frequency and brushing technique, interdental hygiene…)? (N = 205)	
Yes Quite yes Quite no No	9 (4.4) 100 (48.8) 77 (37.6) 19 (9.3)
Do you know that health insurance covers 100% of the cost of an oral examination for pregnant women from their 4th month? (N = 205)	
Yes No	172 (83.9) 33 (16.1)
Have you ever been confronted with a dental problem or question with your patients? (N = 205)	
Yes No	147 (71.7) 58 (28.3)
If so, were you able to respond and/or identify the problem? (N = 147)	
Yes No	73 (49.7) 74 (50.3)

n = number of respondents related to demographic characteristics.

### Knowledge about Oral Health During Pregnancy

Answers to questions about participants’ knowledge are reported in Table 4. 69.3% of midwives surveyed rated their knowledge about the possible link between oral health and pregnancy as insufficient, and 19.5% as non-existent. 198 out of 205 respondents thought that a pregnant woman is more prone to gum disease. 61.5% knew what periodontitis was. However, only 19 midwives correctly defined it. Almost all participants were aware that abscesses and periodontal diseases are risk factors for adverse pregnancy outcomes; incorrectly, 68.3% thought that tooth decay was too. Very few respondents (5.8%) knew that pre-eclampsia can be linked to poor periodontal health. 42.4% wrongly believed that iron deficiency can worsen periodontal disease. Of all the questions asked, no respondent had more than 60% correct answers, and a large majority (73.2%) answered 21%–40% of the questions correctly.

**Table 4:** Knowledge about oral health during pregnancy. n corresponds to number of respondents related to demographic characteristics

Knowledge	Population n (%)
How would you rate your knowledge about the possible link between oral health and pregnancy? (N = 205)	
Very good Good Insufficient Non-existent	1 (0.5) 22 (10.7) 142 (69.3) 40 (19.5)
Do you know what periodontitis is? (N = 205)	
Yes No	126 (61.5) 79 (38.5)
About periodontitis: (N = 126)	
It is a chronic disease It is an inflammatory disease It is an auto-immune disease It can have systemic impacts	28 (22.2) 123 (97.6) 1 (0.8) 83 (65.9)
What do you think are the links between a woman’s oral health and her pregnancy? (N = 205)	
Gum problems are a risk factor for adverse pregnancy outcomes Caries is a risk factor for adverse pregnancy outcomes Pregnancy causes physiological changes of the gums The physiology of pregnancy favors the development of cavities Pregnancy has no effect on the oral condition The oral condition has no impact on pregnancy	132 (64.4) 119 (58.0) 186 (90.7) 88 (42.9) 2 (1.0) 6 (2.9)
Which of the following makes you suspect a risky oral health status? (N = 205)	
Bleeding gums Tooth mobility Yellow teeth Dental plaque or calculus White, brown or black spots on the teeth Halitosis None	154 (75.1) 183 (89.3) 39 (19.0) 93 (45.4) 117 (57.1) 104 (50.1) 1 (0.5)
In your opinion, which oral conditions pose a risk during pregnancy? (N = 205)	
Caries Gingivitis/periodontitis Abscess Food impaction Mouth ulcer None	140 (68.3) 178 (86.8) 199 (97.1) 27 (13.2) 8 (3.9) 0 (0.0)
What are the possible consequences of poor periodontal health on pregnancy? (N = 205)	
Intrauterine growth restriction/hypotrophy Pre-eclampsia Gestational diabetes Imbalance of pre-existing gestational diabetes Threat of preterm delivery Oligoamnios or anamnios Hydramnios Prematurely ruptured membranes Chorioamniotitis Foetal death in utero None	40 (19.5) 12 (5.8) 15 (7.3) 39 (19.0) 165 (80.5) 3 (1.5) 5 (2.4) 118 (57.6) 144 (70.2) 28 (13.7) 15 (7.3)
Do you think a pregnant woman is more prone to gum disease? (N = 205)	
Yes No	198 (96.6) 7 (3.4)
Which factors can promote the progression of the periodontal disease? (N = 205)	
Smoking Iron deficiency Asthma Diabetes Drug use Sinusitis Obesity High blood pressure	201 (98.0) 87 (42.4) 7 (3.4) 174 (84.9) 151 (73.7) 42 (20.5) 82 (40.0) 34 (16.6)

### Current Teachings and Future Needs

The questionnaire focused on two aspects of initial training: first, oral health in general, and second, periodontology (Table 5).

**Table 5:** Current teachings

Training during studies	Population n (%)
Was the importance of oral health discussed during your studies? (N = 205)	
Yes No	58 (28.3) 147 (71.7)
Was the topic of periodontal disease discussed during your studies? (N = 205)	
Yes No	13 (6.3) 192 (93.7)
If yes, when? (N = 13)	
Lecture course Congress Training course	13 (100) 0 0
If yes, how much time was dedicated to this training? (N = 13) < 1 h	
1–2 h > 2 h	8 (61.5) 5 (38.5) 0
If yes, who provided the training? (N = 13)	
A midwife An obstetrician gynecologist A dental surgeon Other	3 (23.2) 3 (23.2) 6 (46.2) 2 (15.4)
Would you like to be trained on the relationship between periodontal disease and pregnancy? (N = 205)	
Yes No	201 (98) 4 (2)

n = number of respondents related to demographic characteristics.

The importance of oral health was discussed during their professional training for only 28.3% of participants. More than half of the graduate midwives trained in Brest responded positively, compared to about 15% for the other training sites (Table 6). Furthermore, there was no statistically significant difference according to practice time. As for periodontal diseases, only 13 midwives had received education on this topic, with less than 1 h devoted to it in most cases.

**Table 6:** Oral health training by location of study

	n	No oral health training n (%)	Oral health training n (%)
Rennes n (%)	83	70 (84.3)	13 (15.7)
Nantes n (%)	12	10 (83.3)	2 (16.7)
Brest n (%)	31	14 (45.2)	17 (54.8)
Abroad n (%)	14	12 (85.7)	2 (14.3)
Totaln (%)		106 (75.7)	34 (24.3)

n = number of respondents related to demographic characteristics.

Finally, 98% of the survey participants answered that they would like to be taught about the relationship between periodontal disease and pregnancy.

## Discussion

Using an online questionnaire, this work aimed to evaluate the knowledge of midwives on periodontal diseases and the involvement thereof in adverse pregnancy outcomes.

The main limitations of this study were a small sample size and the use of an online questionnaire, which poses a possible sample selection bias, as some midwives may use internet applications such as Google Forms more frequently than others. An online questionnaire may also lead to some self-reported bias.

According to the latest statistics from DREES (Direction des Recherches, des Etudes, de l’Evaluation et des Statistiques), the average age of midwives practicing in Brittany is 41.2 years.^9^ One of the survey respondents had practiced for 37.6 years, so the questionnaire distribution via e-mail does not seem to have had a negative impact on the response rate of older midwives. 59.9% of Breton midwives practice at a hospital and 27.2% are self-employed. Although not all midwives responded to the survey, the results were close to this distribution (71.3% and 23.4%, respectively).

Despite the growing number of publications referring to the increased risk of adverse pregnancy outcomes in patients with periodontal disease,^1,4,7,17^ few midwives promote oral hygiene awareness among their patients. In our study, 85.4% of midwives only sometimes or never discussed this topic. As in the study by Boutigny et al,^6^ approximately 30% of midwives recommended very regularly to their patients a check-up with the dentist during pregnancy. However, a study in Australia showed that 39% of respondents regularly discuss oral health with their patients and 49% refer them to a dentist.^31^ In a US study by Naavaal et al, 75% of midwives reported discussing oral health and providing oral health referral to pregnant patients.^29^

These results contrast with the vast majority of midwives surveyed (83.9%) who were aware of the existence of a preventive oral examination for pregnant women, fully covered by the Health Insurance in France. Similarly, Petit et al^35^ showed that although a majority of pregnant women were aware of the potential risk of periodontal disease on pregnancy outcome, only one in five had discussed oral health with a pregnancy professional.

As for the profile of midwives including oral health in their care, there is a significant difference between self-employed midwives and hospital midwives. This could be explained by a longer consultation time for pregnant women in private practice. During pregnancy, patients consulting independent midwives most often visit the same practitioner for different appointments. This avoids redundancy and optimises consultations. Moreover, since self-employed midwives devote most of their activity to consultations, it can be assumed that they are better trained and know how to structure these consultations.

It might also be thought that midwives practicing in level 3 maternity wards would be more willing to inform their patients, given that they are the ones most likely to experience high-risk pregnancies. However, statistical analysis did not confirm this hypothesis. Conversely, the fact that they have had children makes it easier for them to discuss it. One can imagine that on this occasion, they themselves have raised the question of oral health. From these responses, it can be assumed that interest in oral health comes more from personal and professional experience than from initial training.

During hospitalisation for high-risk pregnancies, an infectious disease assessment is often performed to determine the origin of the pregnancy-related pathology. However, oral health assessment does not seem to be part of this check-up since 90.7% of the respondents said they never do it. It would have been interesting to determine what motivated those who responded “sometimes” and “often” and how they were trained. However, these percentages vary from country to country. In Australia and Iran, for example, most midwives regularly perform this examination on their patients.^13,31^ In the Iranian study, more than 80% of midwives routinely referred their patients to a dentist for a check-up.^13^ In an American study, although midwives are well informed about oral health, only 10.3% reported conducting oral health assessments.^29^ This disparity could be explained by a lack of international consensus or a lack of harmonisation in the training of midwives. In this regard, it would have been relevant to include in the questionnaire the possibility for the respondents to specify their personal reasons: possible lack of time, lack of knowledge and/or confidence in the latter, or even lack of priority given to oral health.

As for their ability to provide oral hygiene advice, nearly half of the respondents felt they had little or no ability to do so. In addition, almost all responses (87.9%) were split between “mostly yes” and “mostly no”. These results highlight two problems: the fact that a health care profession in contact with at-risk populations does not know how to give essential oral hygiene advice and the lack of confidence in their knowledge for those who answered positively with “mostly yes” (48.8%). Their own knowledge about the relationship between oral health and pregnancy was rated by 88.8% as non-existent or insufficient, and the results of the multiple-choice questions confirmed these gaps.

Survey participants knew how to recognise the clinical signs of a risky oral condition such as mobility (89.3%) and gingival bleeding (75.1%). However, this knowledge decreased when the questions became more specific, especially regarding periodontitis, with 38.5% not knowing what it is. These results are similar to those of Nguyen et al,^31^ where Australian midwives had only basic knowledge of the subject.

The possible physiological changes of the gingiva during pregnancy seemed to be known by almost all respondents (90.7%) and an even higher percentage (96.6%) knew that a pregnant woman is more susceptible to gum disease. Although the causal link between periodontal disease and adverse pregnancy outcomes seemed to be acquired by the majority of respondents, only the threat of preterm delivery was cited by 80.5% of them. Pre-eclampsia, despite a clearly established link,^40,44^ was mentioned in only 5.8% of the answers. Prematurely ruptured membranes and chorioamniotitis were also cited by a large number of respondents. However, although pathophysiological mechanisms may suggest that periodontal disease plays a role in these disorders, no study has yet proven a correlation.

Pregnancy causes physiological changes that have oral repercussions. Thus, according to studies, 30% to 75% of pregnant women have reported a gravid gingivitis.^2,16,26,45^ However, the topic of oral health has rarely been addressed in the initial training of midwives (28%). As far back as 2013, Egea et al^10^ found that only a very low rate of pregnancy professionals had received teaching on oral pathologies during their initial training. Nearly ten years later, no change appears to have occurred.

There was also a disparity in training between midwifery schools in Western France. In Brest, more than half of the students have been trained on this subject, while this proportion drops to about 15% in Rennes and Nantes.

When responses were analysed by time in practice, there was no significant difference, which was similarly noted by Nguyen et al in their Australian study.^31^ On the other hand, when looking specifically at periodontal disease, only 13 out of 205 respondents had this topic discussed during their studies. It can then be assumed that there is no evolution in training despite the increasing knowledge about the relationship between periodontal disease and adverse pregnancy outcomes.

The vast majority of midwives were aware of their lack of knowledge but seemed to understand the value of being informed on this topic, with 98% expressing a willingness to be trained. This lack of knowledge can be detrimental to pregnant women since this oral risk factor has now been identified. Early screening could allow early treatment of these women. Studies have not been able to demonstrate the effectiveness of non-surgical periodontal treatment on pregnancy outcomes.^25,27,32^ Nevertheless, it is safe and improves the periodontal condition of the pregnant woman. In addition, prevention in expectant mothers has a positive impact not only on pregnancy outcomes but also on the oral health of the children.^42^ Pregnancy is a period when a woman is more receptive to information about her health and is more likely to follow the advice given to her and her family afterwards.

## Conclusions

The results of this study showed that the majority of midwives were not familiar with the correlation between periodontal disease and adverse pregnancy outcomes and did not implement enough screening and prevention. All studies conducted on the same subject agree on the need to improve communication between pregnancy professionals and patients. Midwives should refer all their pregnant patients to a dentist in order not to overlook the ones that are at possible risk for pregnancy complications due to periodontal disease. Multidisciplinary work between the different professions would make it possible to harmonise knowledge and to better detect risky situations.
